# Molecular and life-history effects of a natural toxin on herbivorous and non-target soil arthropods

**DOI:** 10.1007/s10646-012-0861-z

**Published:** 2012-02-05

**Authors:** A. E. Elaine van Ommen Kloeke, Cornelis A. M. van Gestel, Bjarne Styrishave, Martin Hansen, Jacintha Ellers, Dick Roelofs

**Affiliations:** 1 Department of Ecological Science, Faculty of Earth and Life Sciences, VU University Amsterdam, De Boelelaan 1085, 1081 HV Amsterdam, The Netherlands; 2 Department of Pharmaceutics and Analytical Chemistry, Faculty of Pharmaceutical Sciences, University of Copenhagen, Universitetsparken 2, 2100 Copenhagen, Denmark

**Keywords:** 2-Phenylethyl isothiocyanate, Soil dissipation, Ecotoxicology, Gene expression, *Folsomia candida*, *Protaphorura fimata*

## Abstract

Natural toxins, such as isothiocyanate (ITC), are harmful secondary metabolites produced by plants. Many natural toxins occur in commercial crops, yet their possible negative repercussions on especially non-target soil organisms are largely unknown. This study examined life-history and gene transcriptional responses to 2-phenylethyl ITC on two soil arthropod species: *Folsomia candida* and *Protaphorura fimata*. To that end the standardized ISO guideline for ecotoxicological tests and a microarray for *F. candida* were used. The dissipation of 2-phenylethyl ITC in natural soil was investigated using GC-MS/MS for quantification. Half-lives, tested at four concentration levels in natural soil, were on average 16 h with biodegradation as the plausible main removal process. Regardless, toxic effects on reproduction were shown for *F. candida* and *P. fimata*, with EC50 values of around 11.5 nmol/g soil illustrating the toxic character of this compound. Gene expression profiles revealed the importance of fatty acid metabolism at low exposure concentrations (EC10), which is associated with the lipophilic nature of 2-phenylethyl ITC. At higher concentrations (EC50) gene expression became more ubiquitous with over-expression of especially stress-related genes and sugar metabolism. The regulation of a gene encoding a precursor of follistatin, furthermore, implied the inhibition of reproduction and may be an important molecular target that can be linked to the observed adverse effect of life-history traits.

**Electronic supplementary material:**

The online version of this article (doi:10.1007/s10646-012-0861-z) contains supplementary material, which is available to authorized users.

## Introduction

The soil ecosystem is extremely complex and comprises many different organisms, each with their own function, niche and interactions. Soil ecosystem functioning is challenged by many anthropogenic toxins such as metals (He et al. 2005), pesticides (Edwards and Bohlen 1992) and polycyclic aromatic hydrocarbons (Leon Paumen et al. 2009). These compounds may disrupt the soil ecosystem through mortality or reduced reproduction of soil organisms, as was shown by a large number of studies. Natural toxins, on the other hand, are rarely considered a threat to the environment yet can be lethal at low dosages. Natural toxins are organic compounds that are produced as secondary metabolites in fungi, bacteria, algae, plants or animals. Plant toxins (phytotoxins) are used by many edible plants as part of their natural defence system against pathogens and herbivory (Hoerger et al. 2009; van Egmond 2004).

Natural toxins can also be found in the plant family *Brassicaceae*, including broccoli, cabbage and mustard, which all produce glucosinolates (GSL) as secondary metabolites. Tissue damage, due to, for instance, herbivory, causes the enzyme myrosinase to hydrolyse GSL into several possible products of which the most common and most toxic is isothiocyanate (ITC) (Brown and Morra 1997; Halkier and Gershenzon 2006). ITCs have well-known negative effects on several invertebrate species such as fruit flies, wireworms, symphilids and nematodes (Brown and Morra 1997). For instance, 11 μM 2-phenylethyl ITC caused 50% mortality of populations of the root-knot nematode *Meloidogyne incognita* (Lazzeri et al. 2004). Toxic effects are mainly due to irreversible and nonspecific reactions of ITCs with proteins and amino acids, which result in inactivation of enzymes (Brown and Morra 1997).

Most ecological studies involving ITC have focussed on GSL-containing plants and their effects on specific plant pests such as insect herbivores (Hopkins et al. 2009). In contrast, little is known about effects on non-target species; beneficial or neutral species which are unintentionally affected by the toxicant via indirect contact (non-herbivory). Non-target soil organisms can be exposed to ITCs through the decomposition of crop litter, biofumigation or root exudates of GSL-containing plants (Wardle et al. 2004). ITCs do not seem to be target-specific (Brown and Morra 1997) and may, therefore, also affect non-target species. For instance, Jensen et al. (2010) showed a 50% reduction in reproduction of the non-target soil arthropod, *Folsomia fimetaria* when exposed to 65 nmol benzyl ITC per gram soil. Such effects on non-target, but beneficial soil organisms, can have severe negative repercussions on soil functioning.

The present study examined the effect of 2-phenylethyl ITC (the hydrolysis product of 2-phenylethyl GSL) on the survival and reproduction of the non-target fungivorous springtail *Folsomia candida* and the herbivorous springtail *Protaphorura fimata*. Springtails (Collembola) are one of the most abundant soil organisms, providing a significant contribution to nutrient mineralisation and decomposition (Berg et al. 2001; Filser 2002). Moreover, *F. candida* is an important model species for soil quality assessments (Fountain and Hopkin 2005). By complementing measurements on key life-history traits (survival and reproduction) with gene expression profiling (microarray techniques), we also aimed to assess the underlying modes of action of 2-phenylethyl ITC (van Straalen and Roelofs 2008). A microarray for *F. candida* was, therefore, applied to provide insight in the molecular response pathways invoked and the biological processes affected by 2-phenylethyl ITC. Concentrations of 2-phenylethyl ITC in the soil were measured over time, as ITCs are reported to be readily biodegradable (Brown and Morra 1997; Jensen et al. 2010). This is the first report on effects on soil organisms caused by a natural toxin in which measures of survival and reproduction are associated with molecular mechanistic information.

## Materials and methods

### Animals

*Folsomia candida*, a parthenogenetic collembolan (Fountain and Hopkin 2005), was used for both ecotoxicological and microarray experiments. A synchronized culture of either 10–12 days old (ecotoxicological experiments) or 23–25 days old (microarray) animals was prepared from the laboratory stock culture (“Berlin strain”, VU University Amsterdam). This difference in age is chosen due to standardization and aims (reproduction and gene expression effects, respectively) of the existing protocols (ISO 1999; Nota et al. 2009) and the size of the animals at the time of harvesting; microarrays after 2 days and ecotoxicity tests after 28 days. Standardized methods for synchronization and stock maintenance followed the International Standardization Organization (ISO) guideline 11267 (ISO 1999). In short, springtails were kept on moistened plaster of Paris mixed with charcoal, at 20°C, 75% relative humidity and a 12 h light/dark cycle. *Protaphorura fimata*, a herbivorous collembolan (Endlweber et al. 2009), also reproduces parthenogenetically and was cultured in a similar fashion as *F. candida.* This species is, however, difficult to synchronize (Heckmann et al. 2006). Therefore, a method was developed to synchronize animals with a maximum of 1 week difference in age. In short, a large quantity of ‘almost ready to hatch’ eggs (yellow/brown coloured, oval shaped) were carefully transferred from the stock culture to a fresh Petri dish (with a bottom of moist plaster of Paris mixed with charcoal), using a dissecting needle. In order to keep the eggs clean (avoid microbial growth), ~10 adult females were included in this ‘breeding dish’. After 1 week all juveniles were transferred to a fresh Petri dish using a glass Pasteur pipette. Each culture weekly received a fresh quantity of baker’s yeast. To ensure that animals were reproductively active, they were only used for experiments if large quantities of eggs were present in the breeding dish. *P. fimata* used in the toxicity tests were 5–6 weeks old.

### Experimental soil, compound and spiking

For all experiments natural LUFA 2.2 soil (Speyer, Germany) was used, which is a loamy sand soil with a pH of (±SD) 5.5 ± 0.1 and an organic C content of 2.09 ± 0.40%. Before usage the soil was dried at 60°C for 24 h. 2-Phenylethyl ITC [2257-09-2] was obtained from Sigma Aldrich (www.sigmaaldrich.com) as a liquid solution (99% purity). Ten percent of the total amount of soil needed for each treatment was spiked with the desired concentration of 2-phenylethyl ITC, ranging from 3.06 to 20.1 nmol per g dry weight soil, by using stock solutions with acetone as solvent (1:1 ratio, i.e. ml acetone: g dry weight soil, DW) (Brinch et al. 2002). The spiked soils were thoroughly shaken and stored for 24 h in preservation jars to allow the soils to absorb the test chemical. Thereafter, to facilitate complete evaporation of all acetone, jars were left open overnight under a fume hood. Next, the remaining 90% of the total amount of soil needed for each treatment was mixed thoroughly in with the 10% spiked soil. Finally, the soil was moistened to 50% of the water holding capacity of 45.2%, corresponding to 22% water of the soil DW.

### Measuring dissipation in soil

To measure the soil dissipation rate of 2-phenylethyl ITC 5 g samples were taken from the soils spiked with 3.06–4.90–7.84-12.56–20.09 nmol/g soil, representing several concentrations within the test ranges used for the toxicity tests. Time intervals were: dry start (no water added to dry soil), wet start (hydrated soil, time 0), 1, 2, 3, 4, 24, 48, 120, 168 h after initial spiking. The samples were extracted by adding 5 ml ethyl acetate and 100 μl benzyl ITC (500 μmol/l in ethyl acetate) as analytical internal standard (IS) to the samples which were then stored at −18°C in darkness. Prior to analysis, samples were thawed, shaken on a vortex and the ethyl acetate phase was transferred on top of Pasteur pipettes packed with quartz wool inactivated, silica treated) in the bottom and 2.0 g anhydrous Na_2_SO_4_ above to filter and dry the samples. The purified sample eluate was collected in a 10 ml test tube. This procedure was repeated with an additional 5 ml of ethyl acetate added to the initial soil and again transferred to the same Pasteur pipette and eluate into the same test tube. The eluate was then evaporated with nitrogen to nearly dryness (less than 200 μl) and transferred to GC vials. Samples were analysed by gas chromatography tandem mass spectrometry (GC-MS/MS, Varian CP-3800 and Varian 1200 triple-quadruple) with electron ionization (70 eV). The MS was operated in selected reaction ion monitoring mode acquiring data on the ion transitions 163.0 > 105.0 for 2-phenylethyl ITC and 149.0 > 91.0 for benzyl ITC. The GC-column was a 30 m × 0.25 mm, 0.25 μm Factor Four VF-5 MS (Varian). The injection volume was 1 μl in splitless mode achieved by a CTC CombiPAL autosampler and a PTV-injector kept at 200°C at all times. The initial column oven temperature was 40°C and the final temperature was 285°C (15°C/min) with a total analysis time of 18.3 min. Helium was used as carrier gas at 1.0 ml/min. Calibration curves of both benzyl and 2-phenylethyl ITC were used to calculate the amount of 2-phenylethyl ITC present in the soil samples. First order degradation kinetics was used to estimate the degradation half-lives, which was performed in SPSS 15.0.

### Ecotoxicological experiments

Ecotoxicological experiments to determine effects on the survival and reproduction for *F. candida* and *P. fimata* were carried out following the ISO guideline 11267 (ISO 1999). Concentrations of 2-phenylethyl ITC were for the *F. candida* experiment: 3.06-4.90-7.84-12.56-20.09 nmol/g soil and for the *P. fimata* experiment: 3.06-5.51-9.92-17.89-32.16 nmol/g soil. All experiments included a normal control (C) using only LUFA 2.2 soil and an acetone control (AC) with LUFA 2.2 soil spiked with only acetone. Samples were kept in 100 ml jars and consisted of 30 g moist soil, ten animals, a few grains of baker’s yeast and were incubated at 20°C, 70% relative humidity and a 12 h light/dark cycle. Once a week jars were aerated, moisture content was checked and fresh food added. After 28 days 100 ml of water was added to test containers, stirred gently and completely poured out into a glass beaker. For each sample several digital photographs were taken to register all living springtails that came floating to the surface. To establish survival and total number of juveniles, photos were analyzed using the life science microscopy imaging software Cell^D^ (Olympus) (Broerse and van Gestel 2010).

Dose–response curves were calculated to establish lethal concentrations (LC) and effect concentrations (EC, a specific percentage of reduction in juvenile production), using the logistic response model after (Haanstra et al. 1985):

$$ Y(c) = \begin{array}{*{20}c} {Y_{\max } } \\ {\overline{{1 + \begin{array}{*{20}c} x \\ {\overline{(100 - x} )} \\ \end{array} \left( {\begin{array}{*{20}c} c \\ {\overline{{{\text{EC}}_{x} }} } \\ \end{array} } \right)^{b} }} } \\ \end{array} $$

In which *Y* is the percentage of survival (LC_*x*_) or the number of juveniles for (EC_*x*_), with *Y*_*max*_ the estimated maximum number in the untreated control, *x* is the percentage of effect (here either 10 or 50), *c* is the concentration and *b* the slope of the dose–response curve. To investigate if the LC and EC values differed significantly between the two species a generalized likelihood ratio test was performed (Sokal and Rohlf 1995).

### Microarrays experiments

**Fig. 1:**
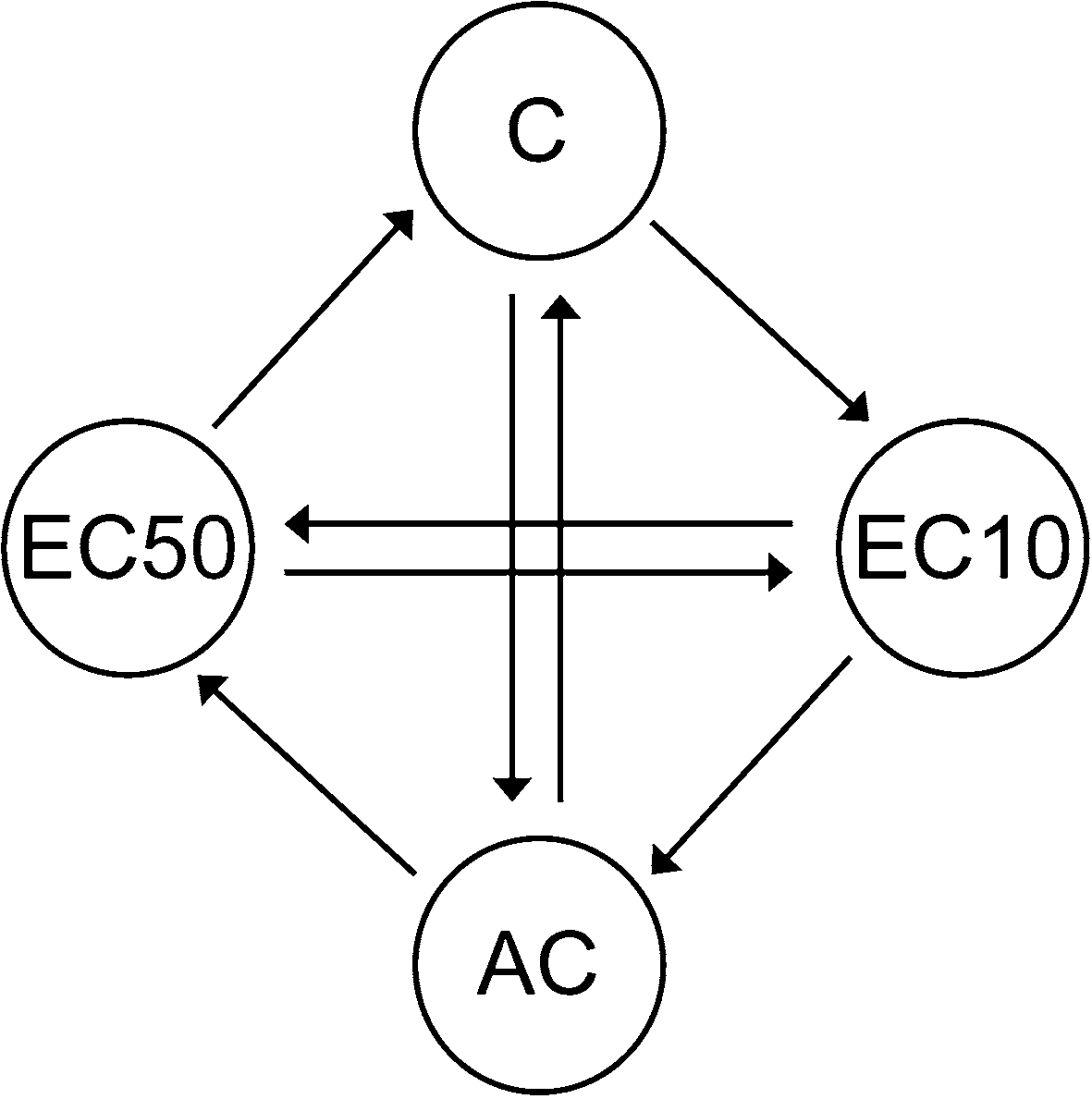
Microarray hybridisation loop design for the four exposure treatments of *Folsomia candida* after 48 h exposure to 2-phenylethyl isothiocyanate in LUFA 2.2 soil. Each treatment had four biological replicates. *C* control, *AC* Aceton Control, *EC10* concentration corresponding to a 10% reduction of reproduction and *EC50* concentration corresponding to a 50% reduction of reproduction. *Arrows* indicate which samples were hybridized together on one two-coloured microarray. *Arrow* direction shows if a sample was labelled with CY3 (green) or CY5 (red): start of arrow = CY3 and end of arrow = CY5

Gene expression analysis was performed for *F. candida* exposed to four treatments including two toxic treatments and the water and acetone control. The two toxic treatments consisted of sub-lethal concentrations of 2-phenylethyl ITC close to EC10 and EC50 values for effects on reproduction (6.21 and 10.7 nmol/g; being the average of the EC10 and EC50 values obtained in the present experiments and in an unpublished pilot experiment). Each treatment had four biological replicates, each consisting of 30 synchronized animals of 23–25 days old. After 2 days exposure, the flotation technique (100 ml demineralised water) was used to harvest animals from the soil. After scooping animals from the water surface with a spoon, they were placed on plaster of Paris to remove excess water. Quickly thereafter animals were placed in a micro-centrifuge tube and snap frozen in liquid nitrogen. RNA preparation, labelling and hybridisation were performed as described by Nota et al. (2009). For hybridisation a loop design was used (Fig. 1). Dyes were swapped at the biological replication level; for each treatment two samples were labelled with the fluorescent dye Cy3 and two with Cy5. A custom made 60-mer, 8 × 15k oligo microarray (Gene Expression Omnibus accession number: GPL7150) based on Agilent technologies was used for this experiment. This microarray contains 5,069 unique probes representing 5,069 different gene clusters from Collembase (www.collembase.org). All probes were spotted randomly in triplicate (Nota et al. 2009).

After feature extraction (Agilent FE software version 9.5.1.1) microarray data was further analysed using the package ‘Limma’ (version 2.18.3 Smyth (2004) in the statistical environment R (version 2.9.0). In short, normexp background correction (Ritchie et al. 2007), LOESS normalization (Smyth and Speed 2003) and aquantile normalization (Hahne et al. 2008) were performed for the whole dataset. As each microarray contained three replicates of the same probe, the consensus correlation, which is a robust average, was taken so that only one data point per microarray remained for each gene (Smyth et al. 2005). Each gene was thus represented by four data points (biological replicates/microarrays). Differential expression was then assessed by means of linear models and empirical Bayes methods. Finally the Benjamini–Hochberg’s false discovery rate method (Benjamini and Hochberg 1995) was used for multiple testing corrections (adjusted *p* < 0.05 was considered significant). MA-plots and boxplots were used for quality control of the data for each array. The expected and observed log ratios of the Agilent spike-in control probes showed a *R* ^2^ > 0.95 for all arrays. Differential expression analysis was performed for several contrasts between the four treatments resulting in a mean log2 expression ratio (treated/untreated) and a *p*-value for each probe on the array. Using Blast2GO (Conesa et al. 2005), part of the differentially expressed genes were annotated to known genes, assigned to a gene ontology (GO) term or referenced to an InterPro number, setting the hit threshold at e-value <1.0e-6. The raw and processed microarray data are available from the NCBI Gene Expression Omnibus (GEO) under accession number GSE29239.

## Results and discussion

### Dissipation of 2-phenylethyl ITC in natural soil

Recovery rates of 2-phenylethyl ITC varied between 52.8 and 78.6% based on the dry start concentrations. Adding water to the soil (dry vs. wet start) caused a significant decrease in the total concentration of 2-phenylethyl ITC in the soil (*p* = 0.03, Student’s *t*-test between measured concentrations of the dry and wet start). A similar effect of water addition was shown by Gimsing et al. (2009) for benzyl ITC, i.e. an increase in water content of the soil increased the degradation rate of benzyl ITC (Gimsing et al. 2009).

**Fig. 2:**
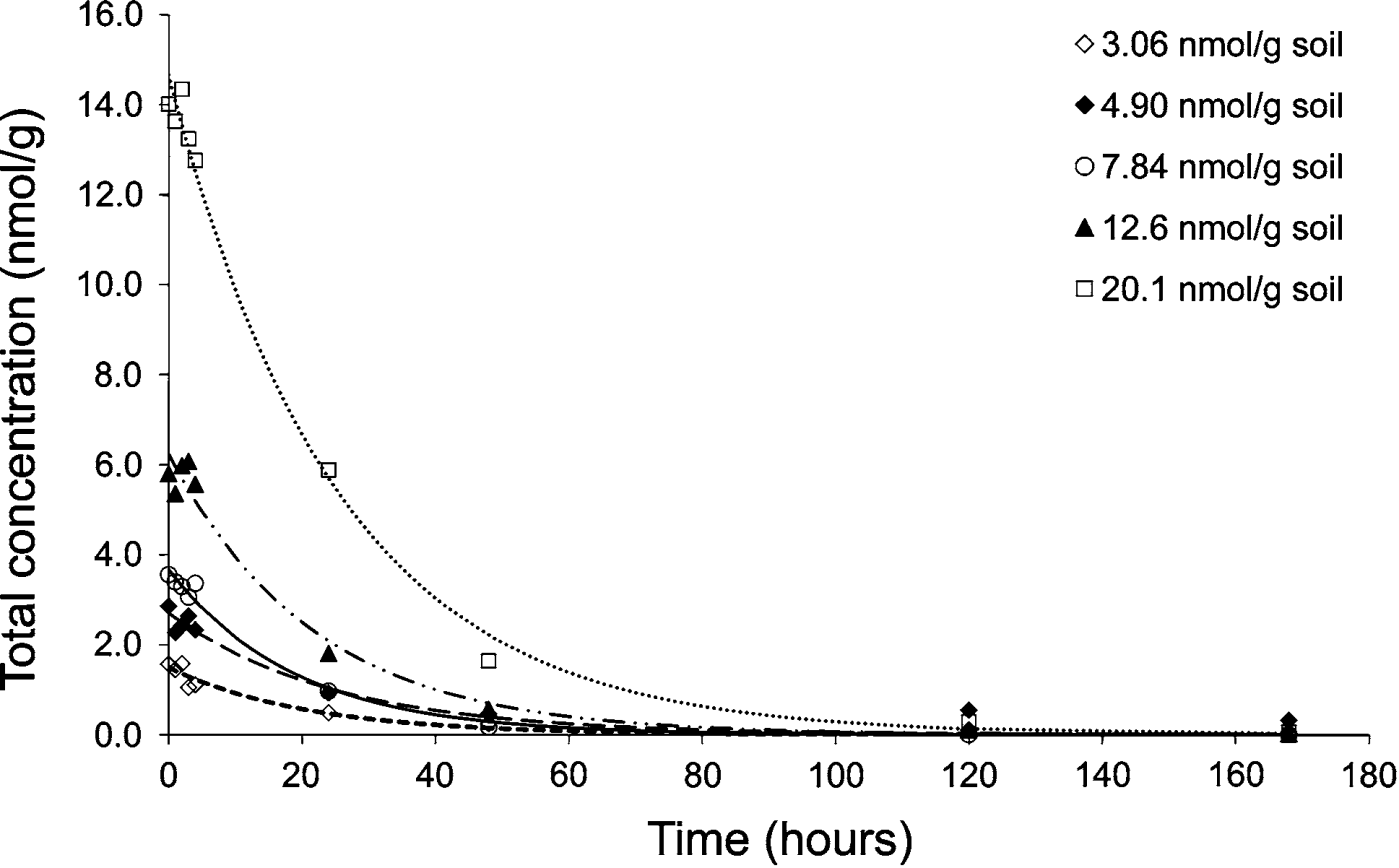
Dissipation of 2-phenylethyl ITC (nmol/g soil) as a function of time (h) in moist LUFA 2.2 soil at 20°C for five different starting concentrations. *Lines* show fits of first-order degradation kinetics

2-Phenylethyl ITC could only be detected in the LUFA soil samples for the first week (Fig. 2), indicating that this compound dissipates rapidly in natural soil. Half-lives were dependent on the starting concentrations and were 14.4 (95% confidence interval: 6.50–22.04) h for 3.06 nmol/g: 17.2 (9.53–24.7) h for 4.90 nmol/g, 13.1 (9.67–16.6) h for 7.84 nmol/g, 15.2 (9.44–20.9) h for 12.6 nmol/g, and 17.6 (14.4–20.8) h for 20.1 nmol/g soil. For the 4.90 nmol/g data set the two last data points (120 and 168 h) were excluded in the half-live model, as these values (still shown in Fig. 2), if included, would yield a very high and different half-life (55.9 h). Jensen et al. (2010) and Gimsing et al. (2009) showed similar dissipation patterns in non-sterile or natural soil for benzyl ITC, a compound with a chemical structure similar to 2-phenylethyl ITC. In sterile soils the dissipation of benzyl ITC was much slower, thus, indicating that microbial degradation is the chief driver responsible for the natural dissipation process of ITCs (Gimsing et al. 2009). We note that the stability of 2-phenylethyl ITC may also be influenced by the presence of test organisms and their associated food source, which was not investigated in this study.

Currently, data on ITC metabolites and their potential toxicity are unknown. Most likely 2-phenylethyl ITC is degraded to benzene, phenol and benzoic acid, which are deemed less toxic than the parent compound. However, as long as the toxicophore (NCS), the ITC part of the molecule, remains intact in any formed metabolite, the molecule is toxic. The potential effects of ITC metabolites, therefore, remain an important issue, which requires attention in future studies.

### Toxic effects on life-history traits

**Fig. 3:**
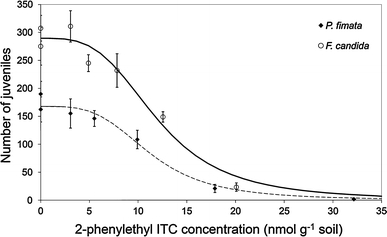
Effects of 2-phenylethyl isothiocyanate (nominal concentrations) on the reproduction of *Folsomia candida* and *Protaphorura fimata* after 28 days exposure in LUFA 2.2 soil. *Lines* show the fit of the logistic dose response model to the data. Error bars are standard errors (*n* = 5)

Toxic effects of 2-phenylethyl ITC on reproduction of both Collembola species are presented in Fig. 3. On average, adult survival of *F. candida* was (±SE) 84 ± 5.1% for the control (C) and 90 ± 0.0% for acetone control (AC) group. Adult survival of *P. fimata* was on average 88 ± 2.0% for C and 90 ± 3.2% for AC. On average *F. candida* produced 275 ± 33.5 juveniles in C and 307 ± 22.9 juveniles in AC. For *P. fimata* the average number of juveniles was 162 ± 22.5 in C and 190 ± 22.6 juveniles in AC. There was no significant difference in survival or reproduction between the two controls for both *F. candida* (survival: *p* = 0.273 and reproduction: *p* = 0.448, student’s *t*-test) and *P. fimata* (survival: *p* = 0.608 and reproduction: *p* = 0.412, student’s *t*-test). Although *P. fimata* is not a common test species for ecotoxicological tests, the species met the validity criteria described by the *F. candida* ISO guideline (1999) with regard to adult survival (mortality in controls <20%) and reproduction (per control vessel >100 juveniles).

2-Phenylethyl ITC proved to be toxic for both collembolan species. At exposure concentrations around 30 nmol/g soil reproduction was completely impaired (Fig. 3). Estimated LC10, LC50, EC10 and EC50 values for both species (based on initial concentrations) are shown in Table 1. It should be noted that the LC and EC-values do not reflect the rapid loss of the compound from the soil. LC and EC values did not significantly differ between *F. candida* and *P. fimata* (Table 1), suggesting that 2-phenylethyl ITC exerts comparable effects on life-history traits of a non-target species as well as an herbivorous pest species. This supports the common assumption that ITCs are not target-specific (Brown and Morra 1997).

**Table 1:** LC- and EC-values for the effects of 2-phenylethyl isothiocyanate on survival and reproduction, respectively of *Folsomia candida* and *Protaphorura fimata* after 28 days exposure in LUFA 2.2 soil. Values are in nmol ITC per gram soil (nominal concentrations)

	*F. candida*	CI	*P. fimata*	CI	χ²
LC10	10.4	(8.67–12.1)	9.32	(6.81–11.8)	0.44
LC50	15.4	(14.1–16.7)	15.2	(13.5–17.0)	0.03
EC10	6.29	(3.73–8.84)	6.08	(2.92–9.24)	0.01
EC50	12.0	(9.94–14.0)	11.2	(8.63–13.8)	0.18

*CI* = 95% confidence interval. Life history trait differences between *F. candida* and *P. fimata* are deemed significant (*p* < 0.05) if χ² > 3.84

Furthermore, 2-phenylethyl ITC is approximately five times more toxic than benzyl ITC of which the EC50 is 65 nmol/g for *F. fimetaria* (Jensen et al. 2010). Apparently, a slight difference in chemical structure (one CH_2_ less) changes the toxicity of ITCs. Many studies show that 2-phenylethyl ITC is among the most toxic ITCs causing adverse effects on a wide range of soil organisms (van Dam et al. 2009). In comparison to anthropogenic toxicants this natural toxin is much more harmful. Fountain and Hopkin (2005) compiled a list of several metals and organic chemicals which were tested with *F. candida* using the ISO guideline (1999). Half of the listed organic chemicals have higher EC50 values than 2-phenylethyl ITC (1.96 mg/kg = 12.0 nmol/g) indicating lower toxicity (Fountain and Hopkin 2005).

The chemical structure of the compound gives insight as to why soil organisms can still come into contact with the toxin; the hydrophobic phenyl group binds firmly to the organic matter of the soil, while the ethyl tail (isothiocyanate), positioned outside soil particles, remains accessible to soil dwelling organisms (Potter et al. 1998). Furthermore, if bioavailable, lipophilic compounds easily bind to lipids, such as the hydrophobic tail region in phospholipid bilayer of cell membranes, which increases the permeability of membranes and contributes in turn to higher contact toxicity (van Dam et al. 2009).

Apparently, even though 2-phenylethyl ITC dissipates rapidly in natural soil and is only present in the soil for a few days, it is able to exert toxic effects on soil arthropods after 28 days. Adult death could be an explanation for reduction in reproduction. In that case the LCx and corresponding ECx should be overlapping. However, confidence intervals only slightly overlapped (Table 1). Reproduction thus also seems impaired in surviving adults on a physiological level, for which gene expression analysis can give more insight.

### Gene expression

Differential expression in *F. candida* was analysed among 5,069 unique genes between control (C), acetone control (AC), versus EC10 and EC50. At both life-history levels, survival and reproduction, the two control groups C and AC did not significantly differ, even though animals in AC seemed to perform better. At the gene expression level, however, a total of 130 genes were significantly differentially expressed between C and AC (for a complete list we refer to Appendix A of the Supporting Information, Table A1). As 2-phenylethyl ITC was spiked into the soil with acetone as solvent, AC was considered the most appropriate control and thus used for further statistical analysis to identify significant transcriptional responses to EC10 and EC50 treatments.

**Fig. 4:**
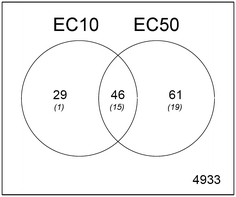
Venn-diagram showing overlap in the total number of significantly differentially expressed genes in *Folsomia candida* after 48 h exposure to 2-phenylethyl isothiocyanate in LUFA 2.2 soil at concentrations corresponding to the EC10 and EC50 for effects on reproduction. Number of annotated genes are represented in *brackets*

The complete list of significantly expressed genes for the comparisons EC10 versus AC and EC50 versus AC can be found in the Supporting Information (Appendix A, Table A2). In the EC10 versus AC comparison, 75 genes were differentially expressed, of which 65 were up-regulated and 10 down-regulated (Table 2). In the EC50 versus AC comparison, 107 genes were differentially expressed, of which 64 were up-regulated and 43 down-regulated. Moreover, 46 genes showed similar differential expression at the EC10 and EC50 level (Fig. 4), so that 29 genes were differentially regulated only at the EC10 and 61 were unique to the EC50. Approximately 30% of all significant genes were annotated using Blast2GO (Conesa et al. 2005). Thirty of the annotated and not-fully annotated differentially expressed genes were placed in the context of biological processes (Table 3) and are discussed in the following sections.

**Table 2:** Total numbers of significantly differentially expressed genes in *Folsomia candida* exposed for 48 h to 2-phenylethyl ITC at concentrations similar to the 28-day EC10 and EC50 for effects on reproduction compared to the acetone control

	EC10	EC50
Up	65	64
	*4994*	*4962*
Down	10	43

Non-differentially expressed genes are given in italics

**Table 3:** Functional grouping of up- or down-regulated genes in *Folsomia candida* after 48 h exposure to 2-phenylethyl ITC in LUFA 2.2 soil at concentrations corresponding to the EC10 (6.21 nmol/g soil) and/or EC50 (10.7 nmol/g soil) for effects on reproduction compared to the acetone control

Process	Gene name	Blast2GO description	E-value	E-code	InterPro	EC10 logFC	EC50 logFC
Fatty acid metabolism							
GO:0006633	Fcc00050^a^	Acetyl-coenzyme a carboxylase alpha	3.32E-82	EC:6.4.1.2		**0.961**	**1.086**
GO:0006629	Fcc01007^a^	Delta-5 fatty acid desaturase	7.10E-34		IPR005804	**0.989**	
GO:0006633	Fcc02959^a^	Fatty acid synthase	4.94E-23	EC:1.3.1.10	IPR000794	**1.082**	**1.266**
GO:0080090	Fcc00142	Sterol regulatory element-binding protein 1	1.43E-15				**0.458**
GO:0045540	Fcc06054^a^	Sec14-like 2 (cerevisiae)	9.79E-09		IPR001251	**0.693**	**0.594**
	Fcc03602	Sec14-like 1 (cerevisiae)	1.85E-15		IPR001251	**0.681**	
	Fcc04835	Sec14-like protein 4 (cral trio domain-containing protein)	1.44E-17		IPR001251	−*1.184*	
	Fcc03980	Diacylglycerol o-acyltransferase	2.44E-09			−*1.554*	
	Fcc03847	Alkaline ceramidase	2.66E-16			**0.643**	
Oxidative stress							
GO:0005515	Fcc00494^a^	Glutathione s-transferase	7.64E-39		IPR004045	**0.703**	**0.830**
GO:0005515	Fcc05973^a^	Glutathione s-transferase	1.48E-42		IPR004045		**1.123**
GO:0055114	Fcc02155^a^	Cytochrome p450	2.84E-32		IPR001128		**1.002**
GO:0005215	Fcc04663^a^	Alpha-tocopherol transfer	4.36E-42		IPR001071		−*0.746*
GO:0006979	Fcc00022^a^	Rec8	1.54E-20		IPR002007		−*1.150*
Immune response							
GO:0009058	Fcc00057^a^	Isopenicillin n synthetase	2.16E-29				**0.856**
GO:0006950	Fcc03623^a^	Advillin	2.71E-47		IPR003128	**0.604**	**0.596**
Sugar metabolism							
GO:0003824	Fcc05611^a^	Trehalase	2.51E-46		IPR001661		**0.868**
GO:0050660	Fcc04789^a^	Glucose dehydrogenase	1.54E-15		IPR007867		**0.827**
GO:0006565	Fcc02499^a^	Cystathionine beta-synthase	5.25E-61	EC:4.2.1.22			**0.788**
GO:0009744	Fcc00529^a^	Maltase- intestinal-like	8.26E-30	EC:3.2.1.26	IPR000322	**1.188**	**1.309**
GO:0015986	Fcc03280^a^	Vacuolar atpase subunit d	5.11E-71	EC:3.6.3.6	IPR002699		−*0.582*
Growth and development							
GO:0005214	Fcc03188^a^	Adult cuticle	3.21E-17		IPR000618		−*0.801*
GO:0005576	Fcc04617^b^	Follistatin precursor	6.66E-29		IPR001239		−*1.351*
	Fcc00558	Chitin binding peritrophin-	4.14E-07			**0.595**	
GO:0006032	Fcc02522^a^	Endochitinase	1.33E-29		IPR000726	**1.110**	**1.080**
GO:0006032	Fcc00881^a^	Acidic mammalian chitinase-like	1.40E-51	EC:3.2.1.0	IPR001223	**1.124**	**0.889**
GO:0008152	Fcc03673^a^	Juvenile hormone acid methyltransferase	2.34E-23	EC:2.1.1.0	IPR013216	**0.794**	**1.034**
Transcription							
GO:0043967	Fcc03584^a^	Dna methyltransferase 1 associated protein 1	9.69E-71				−*0.518*
GO:0003899	Fcc04065^a^	Dna-directed rna polymerase ii subunit rpb4	1.61E-51	EC:2.7.7.6	IPR005574		−*0.716*

Bold indicate up-regulation of genes while italic indicate down-regulation of genes. Empty cells indicate the gene was not differentially expressed compared to the acetone control

*Process* Biological process category and related GO-code of gene (if available), *GeneName* Collembase accession number, *Blast Description* Best Blast2GO hit, *E*-*value* Blast2GO E-value, *E*-*Code* Enzyme Commission number, *InterPro* first code of hits found in InterPro database, *logFC* the log2 transformed ratio (treated/untreated)

^a^ Sequence was fully annotaded by Blast2GO

^b^ Second best annotated Blast2GO description for this sequence

#### Transcriptional response at EC10 level

At the EC10 level, 2-phenylethyl ITC mainly induced metabolic processes (Table 3), especially lipid metabolism, which was indicated by the up-regulation of, for instance, acetyl-coenzyme A carboxylase alpha (Fcc00050), fatty acid delta-6 desaturase (Fcc01007), fatty acid synthase (Fcc02959) and genes encoding for the lipid transport proteins SEC14-like 1 and 2 (Fcc03602 and Fcc06054) (Saito et al. 2007). On the other hand, genes involved in lipid synthesis (diacylglycerol *o*-acyltransferase: Fcc03980) and the binding of small lipophilic molecules (cral-trio domain: Fcc04835), such as 2-phenylethyl ITC, were down-regulated. The cral-trio domain (sec-14 domain) also encompasses SEC 14-like proteins; this domain was thus represented in both the up-regulated and down-regulated genes. It is, however, likely that SEC14-only proteins act as lipid transporters, while the multi-domain SEC14-containing proteins (indicated by cral-trio) have more complex functions, integrating lipid metabolism with other biochemical processes (Saito et al. 2007). Alkaline ceramidase (Fcc03847) was also up-regulated, which codes for an enzyme that is part of a complex bioactive lipid system that mediates cell proliferation, differentiation, apoptosis, adhesion, and migration. It hydrolyses ceramide to sphingosine, a mediator of cell-growth arrest and apoptosis (Mao and Obeid 2008).

Moreover, Fcc00558 was up-regulated. Although not fully annotated by Blast2GO, this sequence had high similarity with a gene encoding for chitin binding peritrophin, which functions in the formation of the extracellular envelope from chitin (peritrophic matrix). This envelope protects the mid-gut of arthropods from physical damage by food particles and from attacks of plant toxins such as 2-phenylethyl ITC (Merzendorfer and Zimoch 2003). Activity of such a process was supported by the up-regulation of genes involved with chitinase (endochitinase: Fcc02522 and acidic mammalian chitinase-like: Fcc00881), an enzyme involved in the moulting process. Taken together, gene expression at the EC10 level revolved around fatty acid metabolism and moulting processes, which can be associated with the lipophilic nature of 2-phenylethyl ITC.

#### Transcriptional response at EC50 level

The effects of 2-phenylethyl ITC on gene expression at the EC50 level were more diverse than the EC10 response (Table 3). Up-regulation of a single cytochrome P450 gene (CYP450: Fcc02155) and two glutathione s-transferases (GST: Fcc00494 and Fcc05973) indicated detoxification by phase I and II biotransformation of 2-phenylethyl ITC to be processed for elimination through the aqueous phase. It is well known that genes coding for CYP450 and GST contribute to the biotransformation of xenobiotics and are oxidative stress responsive as described by their associated GO term (Hayes and Pulford 1995). Interestingly, this process is also activated in humans exposed to 2-phenylethyl ITC and is supposed to be associated with chemoprevention (Cheung and Kong 2010), suggesting that 2-phenylethyl ITC is a xenobiotic compound. Genes associated with vitamin E transport (alpha-tocopherol transfer: Fcc004663), known for its anti-oxidant properties (Dutta-Roy 1999), and oxidation reduction (rec8: Fcc00022) were, however, down-regulated. In addition, isopenicillin N synthase (Fcc00057) and advillin (Fcc03623) were up-regulated. These genes are involved in microbial defence and immune response, suggesting a more general stress response.

Furthermore, 2-phenylethyl ITC affects transcription of genes involved in sugar metabolism in *F. candida*. This is illustrated by the up-regulation of, for instance, trehalase (Fcc05611), glucose dehydrogenase (Fcc04789), cystathionine beta-synthase (Fcc02499) and maltase-intestinal-like (Fcc00529). These transcriptional responses probably serve to increase energy production, which was supported by the down-regulation of vacuolar ATPase subunit d (Fcc03280), an enzyme that converts ATP into ADP. Up-regulation of trehalase was also found as a response of *F. candida* to desiccation stress (Timmermans et al. 2009). Changes in sugar metabolism indicate that organisms invest in many energy-consuming processes, in order to survive stressful conditions.

In addition, a gene encoding the formation of adult cuticle (Fcc03188), important for development and growth, was exclusively down-regulated at the EC50 level. For sequence Fcc04617, the second best Blast2GO hit with a similarity of 79%, coded for a precursor of follistatin. Genes involved with follistatin have been shown to modulate activity of members of the TGF-β super family (bone morphogenic proteins 2 and 4) in vertebrates, which in turn are important for e.g. axis formation, development of the nerve system and embryonic development. In *Drosophila*, follistatin is expressed throughout development where it is, for instance, important for morphogenesis in pupae (Bickel et al. 2008; Pentek et al. 2009). Perturbation of this gene may, therefore, result in failure of early embryonic development and as such overall reduction in reproduction. However, care should be taken to directly extrapolate gene expression to life history effects due to the differences in exposure conditions between ecotoxicological and microarray experiments.

Finally, other gene expression patterns involved the down-regulation of DNA methyltransferase (Fcc03584), mediating DNA methylation, and DNA-directed RNA polymerase ii subunit rpb4 (Fcc04065), involved in RNA synthesis. This suggests that specific parts of DNA, e.g. stress response genes, were more accessible while overall mRNA production was decreased.

#### Overlap in genes between EC10 and EC50

A large overlap was found for gene expression at the EC10 and EC50 treatments (Table 3), which also showed similar direction of regulation (up- or down) for both treatments. Three particular genes, up-regulated in both treatments, are worth mentioning. The first two are genes encoding for endochitinase (Fcc02522) and acidic mammalian chitinase (Fcc00881). Chitinase is an enzyme which is a key constituent of the moulting process of the cuticle and the peritrophic matrix of arthropods (Merzendorfer and Zimoch 2003). Apparently, the moulting process was stimulated as a reaction to the toxin. We speculate that this points towards the lipophilic nature of 2-phenylethyl ITC, resulting in stimulated excretion of epithelium to which the compound is bound. Second, a gene coding for juvenile hormone acid methyltransferase (Fcc03673) was up-regulated in both treatments. Juvenile hormone acid methyltransferase is an enzyme involved in the final step of juvenile hormone synthesis. This group of acyclic sesquiterpenoids regulate many physiological processes such as development, growth, reproduction and diapause (Shinoda and Itoyama 2003). Up-regulation of this gene indicates that these crucial life-cycle processes of *F. candida* are affected by 2-phenylethyl ITC.

## Conclusion

2-Phenylethyl ITC had a noticeable effect on survival and reproduction of both springtail species and the gene expression of *F. candida*. These gene profiles represent hypotheses for explaining the underlying modes of action and acute effects of the toxin on survival and reproduction. For instance, due to the lipophilic nature of the ITC, we observed genes involved in fatty acid metabolism to be severely affected and overall gene activity to be altered. This in turn activated genes encoding biotransformation enzymes, to counteract toxic activity and facilitate excretion of cyclic compounds such as ITC. The regulation of a gene encoding follistatin, furthermore, implied the inhibition of reproduction and may be an important molecular target that can be linked to the observed adverse effect of life history traits. This study clearly shows the detrimental effect of this natural toxin on beneficial non-target and herbivorous springtails. Loss of especially beneficial, functionally important soil invertebrates can result in serious negative repercussions on soil ecosystem functioning (Berg et al. 2001). Natural toxins are present in many agricultural systems and probably at increased levels in the near future. Current interests in ITCs focus on the toxic characteristics that have, for instance, been exploited for alternative pest management methods, so-called biofumigation (Matthiessen and Kirkegaard 2006). Several field and laboratory studies have shown that ITC concentrations can rise up to 100 nmol/g soil after using effective biofumigation strategies (Gimsing and Kirkegaard 2009). Other interests concern the possible chemopreventive nature of ITCs involved with cancer (Traka and Mithen 2009). Such social-economic uses of ITC call for novel commercial health crop varieties with enhanced GSL levels (Traka and Mithen 2009). By definition, as indicated by their secondary metabolite nature, they exert toxicological effects (Hoerger et al. 2009). Potential risks to the environment of these natural toxins, readily available in every-day crops, should therefore be carefully assessed. This study is the first step towards an understanding of these potential risks by studying the effects of a natural toxin at a life-history trait and molecular level.

## Electronic supplementary material

Below is the link to the electronic supplementary material.

Supplementary material 1 All significantly up- or down regulated genes in *Folsomia candida* between control and acetone control treatments exposed for 48 hours in Lufa 2.2.(XLS 55 kb)

Supplementary material 2 All significantly up- or down regulated genes in Folsomia candida exposed for 48 hours to 2-phenylethyl ITC in Lufa 2.2 at concentrations similar to the 28-day EC10 and EC50 for effects on reproduction compared to the acetone control. (XLS 34 kb)
